# Discovery of Anti-PD-L1 Human Domain Antibodies for Cancer Immunotherapy

**DOI:** 10.3389/fimmu.2022.838966

**Published:** 2022-04-04

**Authors:** Hao Liu, Yanli Liu, Zhen Zhao, Yuanke Li, Bahaa Mustafa, Zhijin Chen, Ashutosh Barve, Akshay Jain, Xiaolan Yao, Guangfu Li, Kun Cheng

**Affiliations:** ^1^Division of Pharmacology and Pharmaceutical Sciences, School of Pharmacy, University of Missouri-Kansas City, Kansas City, MO, United States; ^2^Department of Cell and Molecular Biology and Biochemistry, School of Biological and Chemical Sciences, University of Missouri-Kansas City, Kansas City, MO, United States; ^3^Department of Surgery, and Molecular Microbiology & Immunology, School of Medicine, University of Missouri, Columbia, MO, United States

**Keywords:** human single-domain antibody (dAb), phage display, PD-L1, immunotherapy, checkpoint

## Abstract

Immunotherapy using monoclonal antibodies targeting the PD-1/PD-L1 interaction has shown enormous success for various cancers. Despite their encouraging results in clinics, antibody-based checkpoint inhibitors have several limitations, such as poor tumor penetration. To address these limitations of monoclonal antibodies, there is a growing interest in developing low-molecular-weight checkpoint inhibitors, such as antibody fragments. Several antibody fragments targeting PD-1/PD-L1 were recently discovered using phage libraries from camel or alpaca. However, animal-derived antibody fragments may elicit unwanted immune responses, which limit their therapeutic applications. For the first time, we used a human domain antibody phage library and discovered anti-human PD-L1 human single-domain antibodies (dAbs) that block the PD-1/PD-L1 interaction. Among them, the CLV3 dAb shows the highest affinity to PD-L1. The CLV3 dAb also exhibits the highest blocking efficacy of the PD-1/PD-L1 interaction. Moreover, the CLV3 dAb significantly inhibits tumor growth in mice implanted with CT26 colon carcinoma cells. These results suggest that CLV3 dAb can be potentially used as an anti-PD-L1 inhibitor for cancer immunotherapy.

## Introduction

Immunotherapy using checkpoint inhibitors has shown enormous success in treating various cancers. PD-1 is a key inhibitory receptor expressed on immune cells, such as T cells, to restrain autoimmunity by binding to its ligand, PD-L1 or PD-L2 (1). This process is critical for self-tolerance under normal physiologic conditions. However, cancer cells expressing PD-L1 bind to T cells *via* the PD-1/PD-L1 interaction and initiate a negative signaling cascade to inhibit the functions of T cells (2). The PD-1/PD-L1 interaction facilitates tumor growth by dampening the effector T cell-mediated immune response (3). The Food and Drug Administration (FDA) has approved several monoclonal antibodies that bind to either PD-1 or PD-L1 to block the PD-1/PD-L1 interaction. These antibodies have shown considerable success in restoring the killing activity of T cells and improving the antitumor immune response in many types of cancer.

Despite their successful results in clinics, antibody-based checkpoint inhibitors have several limitations. One major disadvantage of antibody-based checkpoint inhibitors is poor tissue penetration due to bivalent binding mechanism and large size (4–6). While effector T cells can migrate deep into solid tumor tissues, antibodies do not readily enter the tumor tissue to achieve a homogenous distribution, which compromises the antitumor efficacy (7). To address this limitation of monoclonal antibodies, there is a growing interest in developing low-molecular-weight checkpoint inhibitors, such as antibody fragments, in the past few years (7–10).

A single-domain antibody (sdAb), also known as VHH or nanobody, is an antibody fragment composed of a single variable domain of heavy-chain only antibodies (HCABs) found in the Camelid family (4). sdAb is the smallest antibody fragment (15 kDa) that maintains the similar antigen-binding affinity as an intact antibody. Since the discovery in 1993, sdAbs have attracted great attention as potential therapeutic and imaging agents, in particular for cancers (11). Compared to intact antibodies, sdAbs have numerous advantages including small size, good stability, ease of production, low immunogenicity, enhanced tissue penetration, and ease of fusion with other proteins (4, 12, 13). In general, sdAbs can quickly and homogenously distribute through tumors compared to antibodies. However, sdAbs also show a rapid renal clearance because of their small size. This could be a disadvantage for sdAb’s therapeutic applications but is a major advantage for using sdAbs as imaging probes or theranostic agents (4, 11, 14). Another disadvantage of sdAbs is their monovalency which leads to relatively lower affinity and blocking efficiency compared to antibody-based checkpoint inhibitors. This limitation can be addressed by constructing a bivalent or trivalent form of sdAbs (15). sdAbs can be discovered through animal immunization or various display libraries, such as phage display, yeast display, bacterial display, and ribosome display (16–18). Several antibody fragments targeting PD-L1 were recently discovered using phage libraries from camel or alpaca (19, 20). However, animal-derived antibody fragments may elicit unwanted immune responses, which limit their therapeutic applications. As a result, there is tremendous interest in developing human sdAbs, also called domain antibodies (dAbs), for therapeutic applications. dAbs can be produced by transgenic mice or phagemid libraries. Compared to natural camelid sdAbs, dAbs are more likely to aggregate due to exposure of hydrophobic heavy chain residues that are normally protected by light chain binding (21, 22).

In the current study, we discover anti-human PD-L1 dAbs using a synthetic human domain antibody phagemid library. The CLV3 dAb exhibits high and specific binding to human PD-L1. It blocks the PD-1/PD-L1 interaction and inhibits tumor growth in mice implanted with CT26 tumor cells. To our knowledge, this is the first anti-human PD-L1 dAb discovered using a synthetic human antibody fragment phage library.

## Methods

### Cell Culture

DU145, MCF-7, 4T1, and CT26 cell lines were purchased from American Type Culture Collection (Manassas, VA). Peripheral blood mononuclear cells (PBMCs) were purchased from iXCells Biotechnologies (San Diego, CA). DU145 cells were cultured in DMEM medium with 10% Fetal Bovine Serum (FBS), 100 units/mL penicillin and 100 μg/mL streptomycin. 4T1 and CT26 cells were cultured in RPMI 1640 medium with 10% FBS, 100 units/mL penicillin and 100 μg/mL streptomycin. MCF-7 cells were cultured in DMEM medium with 10% FBS, 100 units/mL penicillin, 100 μg/mL streptomycin and 1% insulin.

### Phage Display Biopanning and Expression of Discovered dAbs

The Human Domain Antibody Phagemid Library (23) (cat# 6001-hDAb, Source BioScience, Nottingham, United Kingdom) was used to screen anti-PD-L1 dAbs against the recombinant human PD-L1 extracellular domain (ECD) protein (cat# FCL0784B, G&P Biosciences, Santa Clara, CA) as we and others described before (23–25). Briefly, 1×10^12^ pfu phages diluted in PBS containing 5% milk were incubated with human PD-L1 ECD protein immobilized in a 96-well plate at room temperature for 1 h under shaking. Unbound phages were removed by washing with PBS containing 0.1% Tween-20 for ten times. Bound phages were eluted by trypsin and then amplified for the next round biopanning. After four rounds of biopanning, 45 single colonies were randomly selected and sequenced. The sequences encoding dAbs were cloned into the vector pET-22b (+), which was then transformed to E. coli BL21 (DE3) to express dAbs. After lysis of the bacteria using sonication, dAbs were purified using Ni-NTA agarose (cat# P188221, Fisher Scientific) according to the manufacturer’s instruction. The purity of dAbs was assessed by sodium dodecyl sulfate-polyacrylamide gel electrophoresis (SDS-PAGE). To avoid the potential aggregation of dAbs, 5% glycerol was added to dAb solutions (20 mM Tris-base, 500 mM NaCl, pH 8.5). To check potential aggregations, 500 µL of expressed dAbs in Tris buffer (20 mM Tris-base, 500 mM NaCl, pH 8.5) was injected onto a 24 ml Superdex 75 (SD75) size exclusion chromatography column (GE Healthcare) and eluted in the same buffer.

Recombinant proteins expressed in E. coli may be contaminated with lipopolysaccharide (LPS), also known as endotoxin. We, therefore, measured endotoxin in dAbs using the ToxinSensor Chromogenic LAL Endotoxin Assay Kit (GenScript, Piscataway, NJ) according to the manufacturer’s protocol.

### Blockade of the PD-1/PD-L1 Interaction

Streptavidin-HRP (cat# DY998), substrate reagent pack (cat# DY999), and stop solution (cat# DY994) were purchased from R&D Systems (Minneapolis, MN). Ninety-six-well plates were coated with 100 ng of human PD-L1 ECD (cat# FCL0784, G&P Biosciences) or mouse PD-L1 ECD (cat# FCL3502, G&P Biosciences) overnight at 4°C and blocked with 2% BSA for 2 h at room temperature. dAbs were loaded in each well and incubated for 1 h at room temperature. Next, unbound dAbs were removed by washing with PBS. Five hundred nanograms of biotinylated human PD-1 ECD (cat# FCL0761B, G&P Biosciences) or mouse PD-1 (cat# FCL1846B, G&P Biosciences) was loaded in each well and incubated for 1 h. After washing with PBS, 100 µl of streptavidin-HRP (diluted 1:200 in 1% BSA) was added and incubated for 20 min, followed by the addition of substrate reagents and stop solution. The absorbance at 450 nm was recorded with the reference wavelength at 540 nm.

### Binding Specificity to Tumor Cells Overexpressing PD-L1

Binding specificity of dAbs to human PD-L1 was evaluated in PD-L1-positive DU145 cells and PD-L1-deficient MCF-7 cells as described before (24). Cancer cells were detached by non-enzymatic dissociation solution and suspended to 1×10^6^ cells/mL in Opti-MEM medium. Suspended cells were incubated with various concentrations of Cy5-labeled dAbs for 1 h at 37°C with gentle rotation. After washing, the cells were analyzed by flow cytometry.

### Surface Plasmon Resonance (SPR)

Binding affinities of the CLV3 dAb to human PD-L1 ECD and mouse PD-L1 ECD were measured by SPR (BI4500, Biosensing Instrument) at room temperature. PD-L1 ECD proteins were diluted in 10 mM sodium acetate buffer (pH 5.0) and covalently immobilized to a CM5 sensor chip (Biosensing Instrument) using the Amine Coupling Kit (GE Healthcare). A second channel without protein immobilization was used as a reference. Measurements were performed at a flow rate of 60 μL/min in HBS-P+ buffer (GE Healthcare). Various concentrations of dAbs (10, 25, 50, 100, 250, 500, and 2000 nM) were measured to calculate the equilibrium dissociation constant (K_D_). To evaluate non-specific binding, 125 μg/mL BSA was coated on a CM5 chip, and binding affinity of the CLV3 dAb to BSA was determined using the same procedure.

### Cell Apoptosis and Cytokine Release of Co-Cultured PBMCs and DU145 Cells

Cell apoptosis of human PBMCs was evaluated as previously reported (24, 26). Briefly, fresh human PBMCs (1.5×10^5^ cells/well) were cultured alone or co-cultured with DU145 cells (7.5×10^5^ cells/well) in the presence of CLV3 dAb (5 μM) or anti-PD-L1 antibody (1 μM) in 6-well plates at 37°C for 24 h. After incubation, PBMCs were collected and stained with APC Mouse Anti-Human CD3 antibody (cat# 555342, BD Biosciences, San Jose, CA) to gate T lymphocytes. Apoptosis of CD3-positive T lymphocytes was analyzed using the Alexa Fluor^®^ 488 annexin V/Dead Cell Apoptosis kit (cat# A10788, Fisher Scientific) as per the company’s protocol. The supernatant was also harvested for the determination of cytokines including IL-6, IL-10, TNF-α, and IFN-γ using the Bio-Plex Pro assay (Bio-Rad Laboratories, Hercules, CA).

### Penetration in 3D Tumor Spheroids

Penetration of dAbs in 3D tumor spheroids was evaluated as we reported before (24, 27, 28). Tumor spheroids of CT26 cells were formed using the Cultrex 10× Spheroid Formation ECM (Trivigen, Gaithersburg, MD) according to the manufacture’s protocol. CLV3 dAb and anti-PD-L1 antibody were labeled with Cy5 using a Cy5 labeling kit (cat# ab188288, Abcam) according to the manufacturer’s protocol. The tumor spheroids were incubated with 500 nM of Cy5-labeled CLV3 dAb and anti-PD-L1 antibody at 37°C for 6 h. After washing, the spheroids were fixed with formalin, and penetration of the dAb or antibody was assessed using confocal microscopy.

### Animal Study

The animal protocol was approved by the Institutional Animal Care and Use Committee (IACUC) at the University of Missouri-Kansas City. Approximately 5×10^5^ CT26 cells were mixed with an equal volume of matrigel and subcutaneously implanted on the right flank of 5-week-old Balb/c mice (half males and half females). Once the tumor size reached 50-100 mm^3^, the mice were randomly divided into different groups and treated with intraperitoneal injection of dAbs at 5 or 10 mg/kg on a daily basis. The tumor size was calculated with the formula ½×length×width^2^. For anti-tumor activity study, all the mice were euthanized on day 14. For the survival study, the mice were euthanized once their tumors reach 3000 mm^3^. The expression levels of PD-L1, interferon gamma (IFN-γ), and interleukin 6 (IL-6) in tumors were measured using ELISA kits as described before (24). The animal study of dAbs was conducted together with anti-PD-L1 peptides using the same control group (saline) under the same animal protocol. As a result, the saline group was recently reported in our anti-PD-L1 peptide article (24).

### Statistical Analysis

Data are presented as the mean ± standard deviation (SD). The difference between any two groups was determined by one-way analysis of variance (ANOVA) with Tukey’s *post hoc* test. For the animal study, data are expressed as the mean ± standard error of the mean (SEM). P<0.05 was considered statistically significant.

## Result

### Discovery of Anti-PD-L1 dAbs Using Phage Display

**Figure 1:**
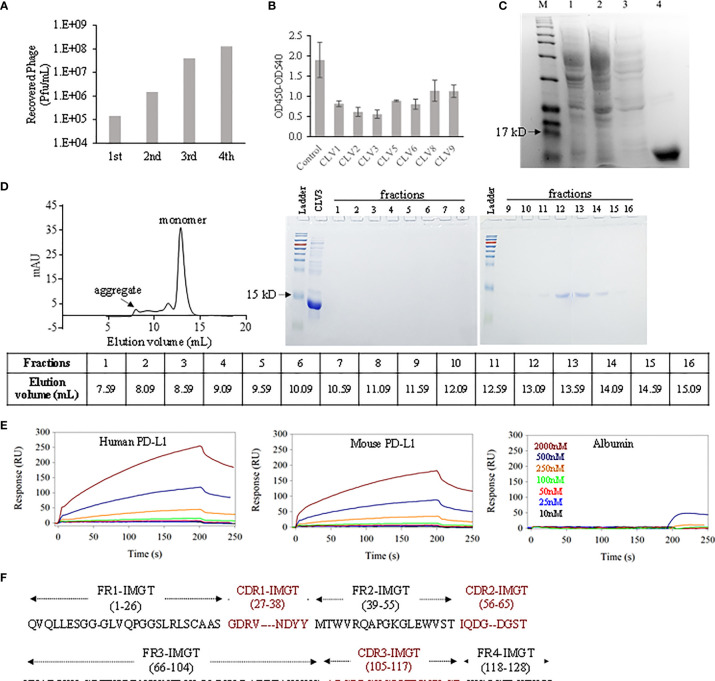
Discovery of anti-PD-L1 dAbs using phage display. **(A)** The number of eluted phages after each round of biopanning. **(B)** Blockade efficiency of the selected dAbs against human PD-L1 ECD at 1 μM. PBS was used as control. **(C)** The CLV3 dAb was expressed in E. coli (BL21 DE3). Proteins in the cell lysate (lane 1), flow-through (lane 2), washing buffer (lane 3), and elution buffer (lane 4) were analyzed using SDS-PAGE. **(D)** Elution profile of the CLV3 dAb on a SD75 size exclusion column. Protein 280 nm absorbance is plotted as a function of the elution volume. Proteins in the elution fractions were evaluated using SDS-PAGE. **(E)** Representative SPR sensorgrams of the CLV3 dAb to immobilized human PD-L1 ECD, mouse PD-L1 ECD, and albumin. The experiment was performed in three replicates. **(F)** The amino acid sequence of the CLV3 dAb.

In order to discover the phage clones that not only bind to PD-L1 but also block the binding between PD-1 and PD-L1, we performed four rounds of biopanning against human PD-L1 ECD instead of whole PD-L1 protein to reduce the possibility of nonspecific binding to the intracellular domain of PD-L1. As shown in **Figure 1A**, we observed a significant enrichment of recovered phages after 4 rounds of biopanning. We randomly selected 45 phage colonies and discovered 9 different sequences. Among them, CLV4 and CLV7 were not selected for protein expression because they contain multiple stop codons in the DNA sequence. The rest 7 DNA sequences were cloned into the vector pET-22b (+), which was then transformed to E. coli BL21 (DE3) to express the encoded dAbs. A PD-1/PD-L1 blocking assay was developed to evaluate the blocking efficacy of these dAbs. The efficiency at which the dAbs block the PD-1/PD-L1 interaction was compared at 1 μM. As shown in **Figure 1B**, all dAbs showed blocking effect. Among them, the CLV3 dAb (amino acid sequence in **Figure 1F**) exhibited the highest blocking ability as it blocked 74% of the human PD-1/PD-L1 interaction. CLV3 was, therefore, selected as the best candidate for following studies because of its high affinity and blocking efficiency.

After purification with Ni-NTA affinity chromatography, the purity of the CLV3 dAb was confirmed with SDS-PAGE (**Figure 1C**). Compared to natural camelid sdAbs, dAbs are more likely to aggregate due to exposure of hydrophobic heavy chain residues that are normally protected by light chain binding (21, 22). We, therefore, used size-exclusion chromatography to monitor potential aggregates in the protein. As illustrated in **Figure 1D**, the major elution peak is at 12.9 mL, consistent with a small dAb monomer. We refer to this peak as P. Two minor peaks at 8.0 mL, 11.5 mL and a slightly increased baseline between these two peaks are also observed, likely due to aggregation and higher molecular weight impurities. We estimated the percentage monomer dAb versus the aggregates and impurities using the area underneath the major peak (P) and the area under the aggregates and impurities (A). The percentage of monomer is calculated using P/(A+P) × 100%, which gives 75%. This value suggests that while some aggregation is present, under our experimental condition, the majority of the protein is a monomer. We used A280 absorbance for the semi-quantitation of the monomer purity. It is worth mentioning that high molecular weight impurities (not aggregation of the dAb) likely have much higher extinction coefficient than the dAb monomer. Therefore, we believe that the purity of the dAb monomer is very likely higher than 75%.

We next measured endotoxin in the CLV3 dAb because recombinant proteins expressed in E. coli may be contaminated with endotoxin, which may affect *in vivo* activity studies. The endotoxin level in the CLV3 dAb was found 0.087 EU/mg, which equals to 0.87 EU/kg for mice in the animal study with a dose of 10 mg CLV3 per kg. According to U.S. Pharmacopeia, the threshold pyrogen dose for humans is 5.0 EU/kg. Therefore, the endotoxin level in the CLV3 dAb is much lower than the threshold pyrogen dose.

### Binding Affinity and Specificity of the CLV3 dAb to Human and Mouse PD-L1

We investigated binding affinities of the CLV3 dAb to human PD-L1 ECD and mouse PD-L1 ECD using SPR. As illustrated in **Figure 1E**, CLV3 shows concentration-depending binding to both human PD-L1 and mouse PD-L1. The K_D_ values of CLV3 to human PD-L1 and mouse PD-L1 are 137.5 and 266.8 nM, respectively. By contrast, the SPR responses of BSA to CLV3 are negligible even at high concentrations of CLV3, suggesting a minimum binding between CLV3 and BSA.

### The CLV3 dAb Blocks the PD-L1/PD-1 Interaction

**Figure 2:**
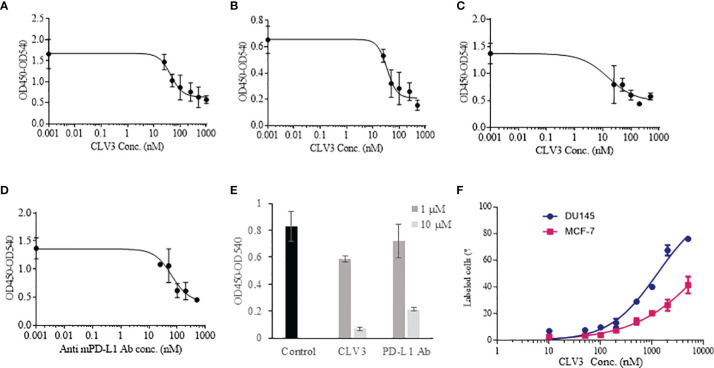
Blocking effect of the dAbs against human and mouse PD-1/PD-L1 interactions. **(A)** Blocking profile of CLV3 against human PD-L1 ECD. **(B)** Blocking profile of CLV3 against PD-L1-positive human prostate cancer DU145 cells. **(C)** Blocking profile of CLV3 against mouse PD-L1 ECD. **(D)** Blocking profile of an anti-mouse PD-L1 antibody (10F.9G2, BioXcell) against mouse PD-L1 ECD. **(E)** Blocking efficiency of CLV3 and the anti-mouse PD-L1 antibody (10F.9G2, BioXcell) against the CD80/PD-L1 interaction. PBS was used as control. **(F)** Binding profile of CLV3 to PD-L1-positive DU145 tumor cells and PD-L1-deficient MCF-7 tumor cells.

Different concentrations of CLV3 were tested to calculate the IC_50_ of CLV3 against the human PD-1/PD-L1 interaction. The IC_50_ of CLV3 was 44.25 nM, with a 70% blocking efficiency (**Figure 2A**). We also evaluated the blocking effect of CLV3 in PD-L1 over-expressing human prostate cancer DU145 cells. The IC_50_ of CLV3 against the PD-1/PD-L1 interaction on DU145 cells was 32.3 nM, and CLV3 blocked 76.5% of the interaction (**Figure 2B**).

Next, we investigated whether CLV3 also block the mouse PD-1/PD-L1 interaction because we need to evaluate the antitumor activity of CLV3 in mice bearing murine cancer cells. As shown in **Figure 2C**, CLV3 blocked approximately 68% of the mouse PD-1/PD-L1 interaction with an IC_50_ of 15.5 nM, which is comparable to its blockade of the human PD-1/PD-L1 (**Figure 2A**). This result is consistent with the binding affinity data (**Figure 1E**) and our previous finding that anti-human PD-L1 peptides exhibit a comparable blocking effect on the mouse PD-1/PD-L1 interaction (24). This is because the PD-1-binding domain of the human PD-L1 shows overall similarity (87.6%) with the mouse protein (29).

The anti-mouse PD-L1 antibody (BioXcell, 10F.9G2) blocked approximately 67% of the interaction with an IC_50_ of 35.2 nM (**Figure 2D**). In our recent work, we found that the IC_50_ of an anti-human PD-L1 antibody is 36.76 nM (24). All these results suggest that CLV3 dAb retains a comparable blocking ability as whole antibodies, although the molecular weight of the dAb is only approximately 15 kD.

In addition to the PD-1/PD-L1 interaction, we also evaluated the blocking effect of CLV3 and anti-PD-L1 antibody on the CD80/PD-L1 interaction. It has been reported that the CD80/PD-L1 interaction partially overlap with the PD-1/PD-L1 and CD80/CTLA4 interfaces. Moreover, the CD80/PD-L1 interaction inhibits T cell activation and cytokine production (30). We, therefore, examined whether CLV3 also blocks the CD80/PD-L1 interaction. As illustrated in **Figure 2E**, CLV3 blocks the CD80/PD-L1 interaction for 29% and 92% at 1 μM and 10 μM, respectively. In contrast, the anti-PD-L1 antibody blocks the CD80/PD-L1 interaction for 13% and 74% at 1 μM and 10 μM, respectively. The result demonstrates that CLV3 exhibited potent blocking efficiency to the PD-1/PD-L1 and CD80/PD-L1 interactions. The specificity of CLV3 to PD-L1 was evaluated in PD-L1-positive DU145 tumor cells and PD-L1-deficient MCF-7 tumor cells (**Figure 2F**). CLV3 showed higher binding affinity to DU145 cells than to MCF-7 cells, suggesting a high specificity of CLV3 to PD-L1 on tumor cells.

### The CLV3 dAb Reduces T Cell Apoptosis Induced by Cancer Cells

**Figure 3:**
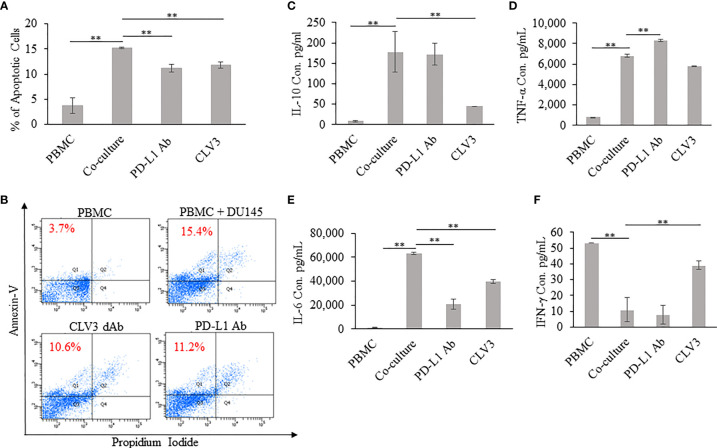
Coculture of human PBMCs and DU145 tumor cells in the presence of the CLV3 dAb. Human PBMCs were cultured alone or co-cultured with DU145 tumor cells in the absence or presence of the CLV3 dAb or anti-PD-L1 antibody for 24 hours. Apoptosis of CD3-positive T cells was evaluated by flow cytometry using Alexa Fluor 488 Annexin V/Dead Cell Apoptosis Kit. **(A, B)** Percentage of apoptotic CD3-positive T lymphocytes. The production of IL-10 **(C)**, TNF-α **(D)**, IL-6 **(E)**, and IFN-γ **(F)** by PBMCs. Results are represented as the mean ± SD (n = 3). (**p < 0.01).

We investigated whether CLV3 inhibits the apoptosis of human PBMCs co-cultured with DU145 cancer cells. As shown in **Figures 3A, B**, the percentage of apoptotic PBMCs increased after co-culture with DU145 cells. The addition of CLV3 to the co-culture cells reduced apoptosis of PBMCs. This is in agreement with a previous report, in which tumor-associated PD-L1 induced the apoptosis of cytotoxic T lymphocytes (26). The authors co-cultured T cells with melanoma cells and found that the tumor cells promoted T cell apoptosis, but apoptosis was not observed in PD-L1 knockout melanoma cells. Moreover, the apoptosis of T cells was significantly reduced after an anti-PD-1 antibody was added into the co-cultured cells (26).

### Analysis of Cytokine Release

The supernatant extracted from 24 h co-cultures of PBMC with DU145 cells was assayed for IL-6, IL-10, IFN-γ, and TNF-α. IL-10 is an inflammatory cytokine that suppresses anti-tumor immune responses by inducing T cell anergy and inhibition of T cell proliferation. As shown in **Figure 3C**, the production of IL-10 is significantly increased in the co-culture of DU145 and PBMC in comparison to PBMC alone. Notably, with the addition of CLV3, the IL-10 level significantly decreased compared to co-culture. Furthermore, it is important to notice that the IL-10 level did not change much with the treatment of the PD-L1 antibody. TNF-α and IL-6 are multifunctional cytokines involved in chronic inflammation and contribution to the progression of cancer. PBMC co-cultured with tumor cells produced high amount of IL-6 and TNF-α than PBMC alone. In comparison, IL-6 and TNF-α secretions are slightly reduced in the presence of CLV3 (**Figures 3D, E**). IFN-γ, a cytotoxic T cells related cytokine, plays an important role in the activation of various cell types such as natural killer cells and T cells. On the other hand, it also inhibits the growth of malignant cells by inducing apoptosis. As shown in **Figure 3F**, co-culture with DU145 significantly decreased the ability of PBMC to express IFN-γ, whereas with the addition of CLV3 there was a dramatic increase in the production of IFN-γ.

### Tumor Penetration of the CLV3 dAb and Anti-PD-L1 Antibody

**Figure 4:**
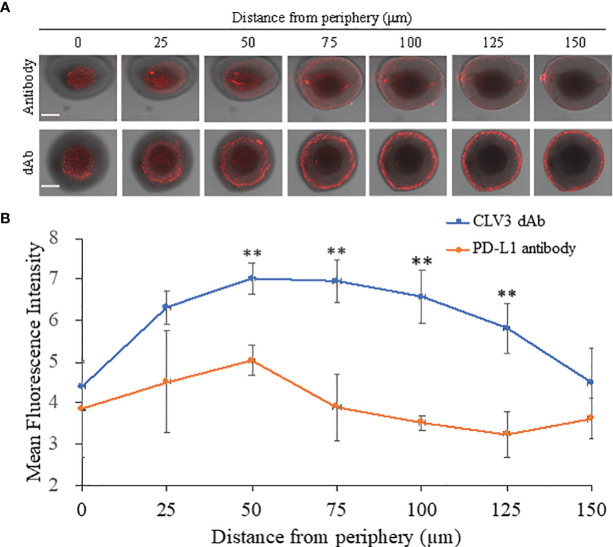
Penetration CLV3 dAb and anti-PD-L1 antibody in 3D tumor spheroids. **(A)** Representative z-stacked confocal images of the spheroids with a z-step of 25 μm after 6 h incubation with Cy5-labeled CLV3 dAb or anti-PD-L1 antibody. The scale bar represents 200 μm. **(B)** Mean fluorescence intensity of the z-stacked images vs. the distance from the periphery of the spheroids. All results are presented as the mean ± SD (n = 3 independent spheroids, **P < 0.01).

A 3D tumor spheroid model of CT26 cells was established to evaluate the penetration of Cy5-labeled CLV3 dAb and anti-PD-L1 antibody. After 6 h of incubation, CLV3 dAb showed a much deeper penetration and higher fluorescence intensity compared with the anti-PD-L1 antibody (**Figures 4A, B**).

### The CLV3 dAb Inhibits the Growth of CT26 Tumor

**Figure 5:**
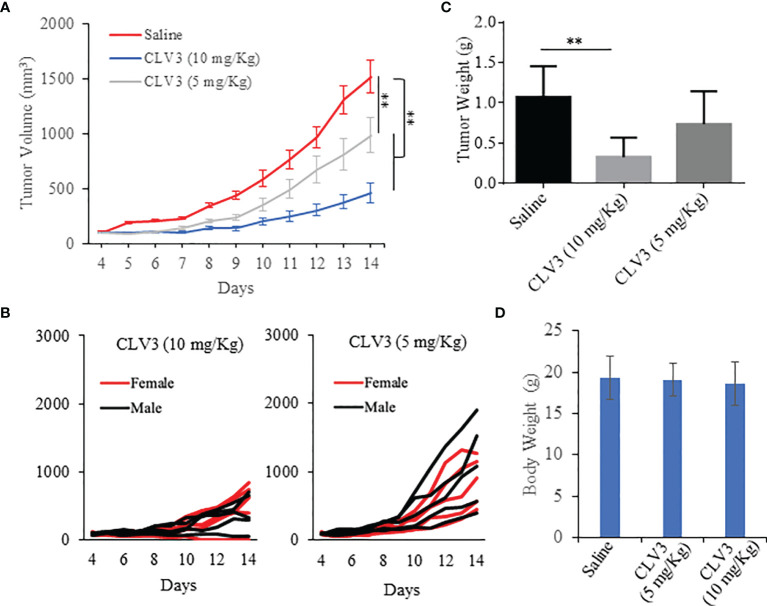
Anti-tumor effect of the CLV3 dAb. Approximately 5×10^5^ CT26 cells were implanted into the right flank of the mice. The treatment was initiated on day 4 when the average tumor volume reached 50-100 mm^3^. CLV3 was administered daily at a dose of 10 mg/kg or 5 mg/Kg for 10 injections. All the mice were euthanized on day 14. **(A)** Tumor growth curve. Tumor volumes are presented as mean ± SEM (n=10). **(B)** Tumor growth curve of individual mice in each group. **(C)** Weight of harvested tumors. **(D)** Body weight of the mice. Results are presented as mean ± SD (n=10). (**p < 0.01).

An animal study was performed to evaluate the anti-tumor effect of CLV3 in mice bearing CT26 mouse colorectal cancer cells as we described before (24, 28). Equal numbers of male and female Balb/c mice were included in each group. As illustrated in **Figure 5**, CLV3 at both concentrations (5 mg/kg and 10 mg/kg) significantly inhibited tumor growth in the mice of both genders. We observed a better inhibitory effect of CLV3 at 10 mg/kg than that at 5 mg/kg on the tumor growth, indicating a dose-dependent response of CLV3. There was no significant difference in the tumor growth between male and female mice (**Figure 5B**). Moreover, we did not observe significant toxicity in the mice, and there is no difference in the body weight of the mice treated with saline and CLV3 (**Figure 5D**).

**Figure 6:**
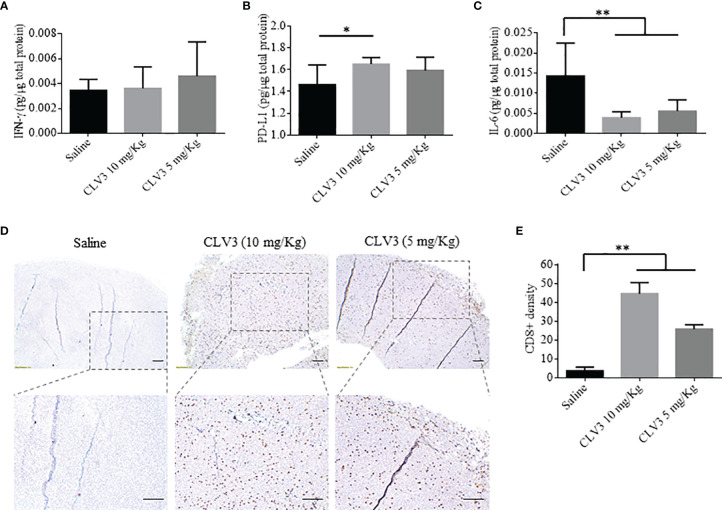
Cytokine expression and immunohistochemical analysis of CD8+ T cells in the tumor. The expression of IFNγ **(A)**, PD-L1 **(B)**, and IL-6 **(C)** in tumors were measured using ELISA. **(D)** Representative images of tumor specimen stained with an anti-CD8 antibody. The scale bar represents 200 µm. **(E)** Quantitation of CD8+ cells in tumor specimen. The results are presented as mean ± SD (n=10). (*p < 0.05, **p < 0.01).

Next, we measured the expression of IFN-γ, PD-L1, and IL-6 in the tumor. IFN-γ is a multifunctional cytokine in cancer progression. While IFN-γ exhibits antitumor immunity by inducing Th1 polarization, it also induces PD-L1 expression on tumor cells to escape immune attach (31). Direct contact with CD8+ T cells or secreted IFN-γ induces the expression of PD-L1 on tumor cells (32). For example, knocking down the IFN-γ receptor in an ovarian mouse model decreases PD-L1 expression on tumor cells and increases the infiltration of CD8+ T cells, leading to prolonged survival of the mice. The administration of IFN-γ into the tumors induces PD-L1 expression and promotes tumor growth. IFN-γ secreted by CD8+ T cells upregulates the expression of PD-L1 on cancer cells and subsequently increases tumor growth (33). The PD-L1 expression on tumor cells is induced by the secreted IFN-γ present in the tumor microenvironment (31). As illustrated in **Figure 6A**, treatment with CLV3 dAb does not significantly change the expression of IFN-γ in tumor tissues. By contrast, CLV3 dAb increases the PD-L1 expression at the higher dose of 10mg/kg (**Figure 6B**). This result is consistent with previous reports (34, 35).

The treatment with CLV3 dAb decreases the IL-6 level in tumors compared to the saline group (**Figure 6C**). This is in accordance with a report showing a decline in IL-6 levels in cancer patients treated with the anti-PD-L1 antibody Atezolizumab (36). In another study in murine models of pancreatic cancer, the blockade of IL-6 using an antibody enhances the antitumor efficiency of anti-PD-L1 antibodies. This is because the IL-6/STAT3 pathway favors the immunosuppressive cells and dysregulates the T cell subsets, such as myeloid-derived suppressor cells (MDSCs) and T regulatory cells, leading to tumor progression (37).

We also compared the density of CD8+ T cells in tumor tissues by immunohistochemistry. CLV3 dAb significantly increases the density of CD8+ cells in tumors in a dose-dependent manner (**Figures 6D, E**). This is in agreement with studies showing the augmentation of CD8+ T cells in patients who responded to anti-PD-1 antibody therapy. Meanwhile, CD8+ T cells were not detected in patients who did not respond to the treatment (34, 36). In another study, Curran et al. reported a significant surge in CD8+ T cells in a B16 melanoma tumor model after treatment with an anti-PD-L1 antibody and Fvax vaccination (38).

**Figure 7:**
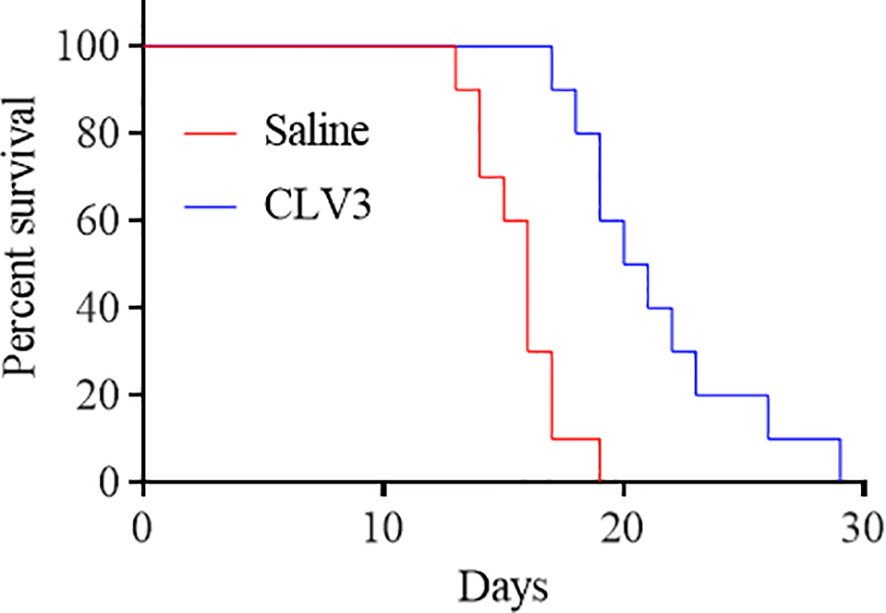
The anti-PD-L1 dAb prolongs the survival of mice bearing CT26 tumors. CT26 tumor-bearing mice were randomly divided into two groups (10 mice per group, 50% female and 50% male). The mice were treated with the CLV3 dAb at a dose of 10 mg/kg from day 4 to day 17. The mice were euthanized once their tumors reach 3000 mm^3^.

We next investigated the survival of CT26 tumor-bearing mice. The mice were treated with CLV3 at a dose of 10 mg/kg daily from day 4 to day 17 post-tumor implantation. As shown in **Figure 7**, the treatment increased the survival rate of mice compared to the saline group. By day 17, most of the mice in the saline group died, but only one mouse in the CLV3 treated group died. Overall, CLV3 significantly increased the survival rate of the tumor-bearing mice.

## Discussion

Currently, three anti-PD-1 and three anti-PD-L1 antibodies have been approved by the FDA. The antibodies block the PD-1/PD-L1 interaction and subsequently restore the immune cell killing ability (3, 39). However, blockade of the PD-1/PD-L2 interaction by antibodies stimulates tumor-promoting Th2 inflammation, implying that the unwanted PD-L2 blockade would be a side effect of anti-PD-1 antibodies but not the anti-PD-L1 antibodies (40, 41). PD-L1-specific blockade would be preferred to PD-1-specific blockade (19). Compared to traditional intact antibody, sdAb, the smallest naturally occurring antibody fragment in Camelid family, retains a comparable blocking ability as whole antibodies (15). sdAbs have numerous advantages, including small size, low immunogenicity, and easy production. In addition, sdAbs are also featured with relative short retention time in the circulation. However, although the circulation half-life of dAbs is just about 30 min to 2 h, researchers discovered that dAbs can bind to target proteins (i.e., CD47) after 24 hours (42, 43). sdAb can also carry payloads (e.g., radioisotopes and cytokines) to the tumor and reduce the systemic exposure because of their rapid systemic clearance but sustained half-life in target tissues. In addition, the production of sdAb is much easier than monoclonal antibody. sdAb can be easily expressed in bacteria with a high yield, which makes the sdAb a good candidate for the scaling up production (18). Thus, sdAb represents a new type of checkpoint inhibitors for cancer immunotherapy. For example, promising results from one phase III clinical trial of a sdAb (caplicizumab) targeting acquired thrombotic thrombocytopenic purpura (aTTP) were reported in 2017 (44), and it was approved by the FDA in 2019 as the first nanobody-based drug.

Despite the success of sdAb in clinical trials and other applications, it is worth mentioning that sdAb has several inherent disadvantages that need to be considered during the drug development of sdAbs. In general, sdAbs show a rapid renal clearance because of their small size. This is a disadvantage for sdAb’s therapeutic applications but is a major advantage for using sdAbs as imaging probes or theranostic agents (4, 11, 14). Another disadvantage of sdAbs is their monovalency which reduces their affinity and blocking efficiency compared to antibodies. This limitation can be addressed by constructing a bivalent or trivalent form of sdAbs. For example, the bivalent and trivalent format of a sdAb are 313- and 135-fold more potent, respectively, than the monovalent format (15).

In the current study, we discovered 7 different dAbs that blocked the PD-1/PD-L1 interaction between tumor cells and immune cells. Among all these candidates, CLV3 showed the best binding affinity to PD-L1 and the most potent ability to block the PD-1/PD-L1 interaction. We evaluated the blocking effect *in vitro* and *in vivo*.

CLV3 successfully inhibited tumor growth and increased the survival rate of tumor-bearing mice compared to the control group. We observed significantly increased CD8+ T cell numbers in tumor tissues after treatment. The IFN-γ, PD-L1 and IL-6 levels were also detected. The CLV3 increased the IFN-γ and PD-L1 levels and decreased the IL-6 level. As the previous reported (34), the PD-L1 expression was upregulated after the treatment. The PD-L1 level was associated with the CD8 density in the tumor tissue. The researchers suggested that the CD8 detection directly influenced the therapeutic outcome during the immunotherapy. Consistent with the previous report, we observed a significant tumor inhibition, correlated with statistically elevated CD8 detection in the tumors. Thus, we suggest the CLV3 dAb represents a potentially anti-PD-L1 inhibitor for cancer immunotherapy. Construction of a bivalent or trivalent form of the CLV3 dAb can further increase its activity. Moreover, the CLV3 sdAb can be easily used to construct bispecific inhibitors to simultaneously target two different checkpoint pathways.

## Data Availability Statement

The original contributions presented in the study are included in the article/supplementary material. Further inquiries can be directed to the corresponding author.

## Ethics Statement

The animal study was reviewed and approved by UMKC IACUC.

## Author Contributions

HL: Data curation, Formal analysis, Investigation, Methodology, and writing-original draft preparation. YKL: Data curation, Formal analysis, Investigation, Methodology, and writing-original draft preparation. ZZ and YKL: Help in the *in vitro* activity studies. BM, AB, and AJ: Help in the animal studies. XY: Help in the expression of dAbs. Review and Editing. GL: Writing-Review and Editing. KC: Conceptualization, Funding acquisition, Project administration, Supervision, Validation, Writing-Review and Editing. All authors contributed to the article and approved the submitted version.

## Funding

This work was supported in part by the National Institutes of Health (grant number R01AA021510 and R01CA231099).

## Conflict of Interest

The authors are in the process of filing a patent of the dAbs discovered in this study.

## Publisher’s Note

All claims expressed in this article are solely those of the authors and do not necessarily represent those of their affiliated organizations, or those of the publisher, the editors and the reviewers. Any product that may be evaluated in this article, or claim that may be made by its manufacturer, is not guaranteed or endorsed by the publisher.
